# Re-challenging immune checkpoint inhibitor in a patient with advanced non-small cell lung cancer: a case report

**DOI:** 10.1186/s12885-018-4212-1

**Published:** 2018-03-20

**Authors:** Taiki Hakozaki, Yusuke Okuma, Jumpei Kashima

**Affiliations:** 1 Department of Thoracic Oncology and Respiratory Medicine, Tokyo Metropolitan Cancer and Infectious Diseases Center Komagome Hospital, 3-18-22 Honkomagome, Bunkyo, Tokyo, 113-8677 Japan; 2 Department of Pathology, Tokyo Metropolitan Cancer and Infectious Diseases Center Komagome Hospital, Tokyo, 113-8677 Japan

**Keywords:** Nivolumab, Immune checkpoint inhibitor, Non-small cell lung cancer, Immune-related adverse events, Re-challenge

## Abstract

**Background:**

Currently, immune checkpoint (ICP) inhibitors are essential drugs for the treatment of non-small cell lung cancer (NSCLC). However, in patients previously treated with ICP inhibitors, the efficacy and safety of re-challenging the same or another ICP inhibitor remain unclear.

**Case presentation:**

We present the case of a patient treated with nivolumab for advanced NSCLC who was previously treated with an ICP inhibitor as the first-line chemotherapy along with heavy cytotoxic chemotherapy. After the failure of five lines of chemotherapy, 3 cycles of nivolumab, as the ICP inhibitor re-challenge, the patient achieved a partial response.

**Conclusions:**

This case might suggest that re-challenging an ICP inhibitor could be clinically active in selected patients with advanced NSCLC who progress after achieving an initial clinical benefit with an ICP inhibitor.

## Background

Immune checkpoint (ICP) inhibitors, including nivolumab, pembrolizumab, and atezolizumab, are currently approved for advanced non-small cell lung cancer (NSCLC). The CheckMate-017 [1], CheckMate-057 [2], KEYNOTE-010 [3], and OAK [4] trials demonstrated the clinical benefit, as well as the long-tailed effect, of these agents over docetaxel, which was the standard of care (SoC) in the second-line therapy. The KEYNOTE-024 [5] demonstrated the prolonged progression-free survival (PFS) with pembrolizumab over platinum doublet chemotherapy in the first-line setting. Reportedly, immune-related adverse events (irAEs), which occur in approximately 20% of patients, are the leading adverse events related to ICP inhibitors [2]. Once high-grade irAEs occur during treatment with ICP inhibitors, clinicians are required to discontinue the use of the ICP inhibitors. In a majority of cases, the resolution is achieved with corticosteroids. However, re-initiation of ICP inhibitors is often challenging because further anti-PD-1/PD-L1 are required to obtain the best durable disease control. To date, limited data are available about the efficacy and safety of re-challenging ICP inhibitors. Furthermore, the median PFS of patients with advanced NSCLC treated with ICP inhibitors is approximately 3–4 months [2], although some patients achieve a long-lasting response with ICP inhibitors. At present, cytotoxic chemotherapy is the SoC for patients after the disease progression with ICP inhibitors. Nonetheless, the efficacy and safety of re-challenging the same or another ICP inhibitor in such settings remain unclear.

Herein, we present the case of a patient with advanced NSCLC who was previously treated with the first-line ICP inhibitor and demonstrated the clinical response to nivolumab as the sixth-line treatment receiving cytotoxic chemotherapy.

## Case presentation

**Fig. 1:**
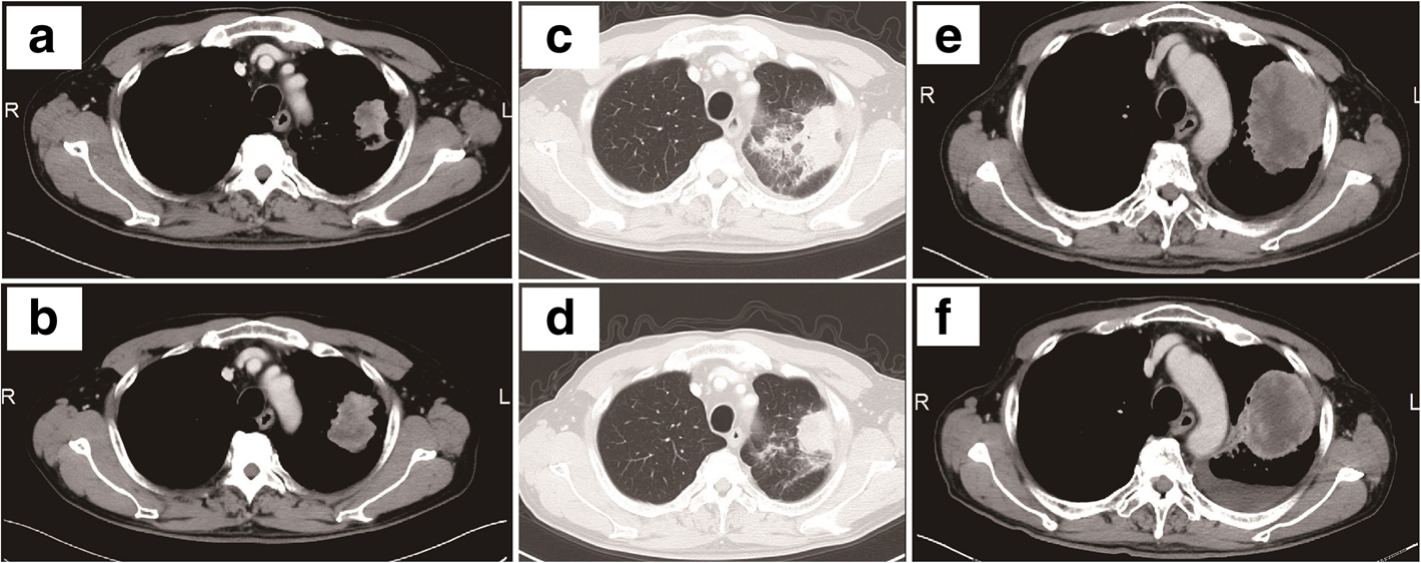
Radiographic results before and after ICP inhibitors. The CT scan shows the primary lesion in the right upper lobe before the first-line ICP inhibitor (**a**), SD after 2 months accompanied by alveolitis (**c**, **d**), and enlarged after 4 months (**b**). After the failure of five lines of chemotherapy (**e**), the primary lesion responded to 3 cycles of nivolumab (**f**)

**Fig. 2:**
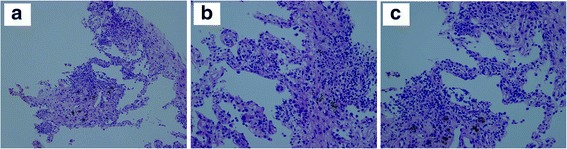
Lung biopsy demonstrated mild fibrinolytic hyperplasia of alveolar septum and strong infiltration of lymphocytes, including small fraction of eosinophil. Alveolar cells were swollen and form cells were accumulated ((**a**): X100, (**b**, **c**): X200)

A 72-year-old Japanese male presented with an abnormal chest opacity that was determined to be adenocarcinoma of the left upper lobe at cT2aN1M1b Stage IV, without epidermal growth factor receptor (EGFR) mutation and anaplastic lymphoma kinase (ALK) translocation (Fig. 1a). The patient was a never smoker with no specific medical history, except for duodenal ulcer, appendicitis, and hypertension. His Eastern Cooperative Oncology Group (ECOG) performance status was 0. He was enrolled in the clinical trial and was randomized to receive a PD-1 inhibitor (an investigational new drug) as the first-line treatment. After 3 weeks of his second cycle, he presented with a productive cough. A computed tomography (CT) scan revealed an infiltrative shadow and the ground glass opacity around the primary lesion in the left upper lobe (Fig. 1c). In addition, transbronchial lung biopsy suggested drug-induced alveolitis, which was considered as grade 2 irAE caused by the ICP inhibitor (Fig. 2). Accordingly, we administered and down-titrated oral prednisolone (25 mg/day), which reduced alveolitis to grade 1 after 2 weeks and normalized by 6 months. After remaining at a stable disease (SD) for 2 months (Fig. 1d), the restaging CT scan of the patient at 4 months revealed an enlarging primary tumor (Fig. 1b). He was subsequently treated with cytotoxic agents, such as cisplatin, pemetrexed, docetaxel, S-1, and nanoparticle albumin–bound paclitaxel, all of which ended with the disease progression (Fig. 1e). After that, the patient was treated with nivolumab (2 mg/kg, day 1, every 2 weeks) as the sixth-line therapy. After 6 weeks of initiating nivolumab treatment, a CT scan revealed a partial response in the primary lung lesion (Fig. 1f). After 6 cycles of nivolumab, the routine imaging surveillance of the patient revealed no disease progression and no irAEs, including recurrence of alveolitis.

## Discussion and conclusions

Here, we reported the case of a patient who responded to nivolumab in the later line of treatment, which had previously failed with a PD-1 inhibitor and cytotoxic chemotherapy. Although the patient experienced irAE with the PD-1 inhibitor treatment, the recurrence of irAE was not observed in nivolumab treatment. To date, limited published data is available about the efficacy and safety of re-challenging ICP inhibitors. To the best of our knowledge, no study to date has elucidated the clinical benefit of re-challenging an ICP inhibitor in patients with advanced NSCLC.

In patients with metastatic melanoma, some studies have reported the efficacy and safety profile of retreatment with ipilimumab, a fully monoclonal antibody against cytotoxic T-lymphocyte-associated antigen-4 (CTLA-4), or a combination of nivolumab and ipilimumab, after an initial period of disease control (Table 1) [6–8]. Regarding the efficacy, re-challenging ICP inhibitors achieved a relatively favorable response. For patients who were retreated with ipilimumab after the initial failure with ipilimumab, the overall response rate (ORR) and disease control rate (DCR) were 11.8–23.0% and 48.4–60.5%, respectively. In contrast, for patients who were only retreated with nivolumab after the initial failure with a combination of ipilimumab and nivolumab, the ORR and DCR were 70.0 and 88.8%, respectively [6, 7, 9, 10]. In these studies, the response of some patients to ipilimumab improved upon re-challenging compared with induction, implying that the re-challenge with ipilimumab induced renewed or even deeper antitumor activity, although the precise mechanism remains poorly understood. In this case, the patient’s immunity against the tumor, which shifted to “escape” phase at the time of pembrolizumab therapy failure, might have been reactivated by any changes during subsequent treatments. Possible justifications for the transition in the responsiveness to ICP inhibitors include changes in the (1) tumor mutation and neoantigen load, (2) tumor-infiltrating T-cell repertoire, and (3) immunosuppressive tumor microenvironment. In our patient, such changes might have been induced by previous ICP inhibitor and cytotoxic chemotherapy exposure or disease progression itself. Remarkably, a recent research of the T-cell repertoire demonstrated the association of responses to nivolumab with different patterns of the T-cell diversity dynamics according to previous ipilimumab exposure [11].

**Table 1:** Retreatment with ICP inhibitors in metastatic melanoma

	Ipi → Ipi			Ipi + Nivo→Nivo
	Robert et al. [5] (*n* = 38)	Chiarion-Sileni et al [7] (*n = 51*)	Lebbe et al [10] (*n* = 122)	Pollack et al [8] (*n* = 80)
ORR (%)	18.4	11.8	23.0	70.0
DCR (%)	60.5	54.9	48.4	88.8
All grade irAE (%)	57.9	21.6	64.0	50.0
Grade 3/4 irAE (%)	10.5	13.5	13.5	30.0

*Ipi* ipilimumab, *Nivo* nivolumab, *ORR* overall response rate, *DCR* disease control rate, *irAE* immune-related adverse event

Regarding safety, ipilimumab retreatment was well tolerated [6–10], and any grade irAEs and grade 3 or 4 irAEs were observed in 21.6–60.4% and 5.9–30.0%, respectively (Table 1). In addition, the frequency of treatment-related irAEs during retreatment was similar to those observed during induction and was manageable with established algorithms used in induction immunotherapy. A study suggested that the type of toxicity in induction immunotherapy, the absence of steroids at re-challenge, and the interval before re-challenge could be potential predictors of recurrent or novel severe toxicities, whereas the severity of initial toxicity or the duration of immunosuppression demonstrated little correlation [7].

In a prior case series focusing on patients who developed pneumonitis associated with PD-1/PD-L1 inhibitors, three among twelve (25%) patients who underwent re-challenge with ICP inhibitors after an initial pneumonitis event experienced recurrent pneumonitis, which was resolved in all with corticosteroids or ICP inhibitor discontinuation [12]. Interestingly, some patients experienced recurrence of pneumonitis after initial clinical improvement without re-challenge of ICP inhibitors.

In addition, recent studies have highlighted the correlation of the development of irAEs with better clinical outcomes of ICP inhibitors treatment in NSCLC as well as melanoma [13–15]. The CheckMate-153 trial represented the prolonged PFS of patients with NSCLC receiving the continuous nivolumab treatment compared to those who discontinued within a year [16]. The increment in the incidence of irAE is proportional to the duration of ICP inhibitors treatment, raising the conflict about the efficacy of ICP inhibitors re-challenge for patients with NSCLC. Hence, further research is warranted to establish the optimal sequence of treatment, including the consideration for ICP inhibitors re-challenge based on these insights. At present, with little evidence on efficacy and safety of ICP inhibitors in patients with advanced NSCLC, ICP inhibitors require deliberation on the risk–benefit of re-challenging on the individual basis with adequate informed consent.

This case might suggest the potential efficacy of re-challenging ICP inhibitors in selected patients with advanced NSCLC who progress after achieving initial clinical benefit with ICP inhibitor treatment. Nevertheless, further investigation is warranted to validate the efficacy and safety of re-challenging ICP inhibitors in patients with NSCLC.

## Abbreviations

CT: Computed tomography
irAEs: Immune-related adverse effects
NSCLC: Non-small cell lung cancer
PD-1: Programmed death receptor-1
PD-L1: Programmed death receptor ligand 1;
SoC: Standard of care

## References

[1] Brahmer J, Reckamp KL, Baas P, Crino L, Eberhardt WE, Poddubskaya E, Antonia S, Pluzanski A, Vokes EE, Holgado E (2015). Nivolumab versus docetaxel in advanced squamous-cell non-small-cell lung Cancer. N Engl J Med.

[2] Borghaei H, Paz-Ares L, Horn L, Spigel DR, Steins M, Ready NE, Chow LQ, Vokes EE, Felip E, Holgado E (2015). Nivolumab versus docetaxel in advanced nonsquamous non-small-cell lung Cancer. N Engl J Med.

[3] Herbst RS, Baas P, Kim DW, Felip E, Perez-Gracia JL, Han JY, Molina J, Kim JH, Arvis CD, Ahn MJ (2016). Pembrolizumab versus docetaxel for previously treated, PD-L1-positive, advanced non-small-cell lung cancer (KEYNOTE-010): a randomised controlled trial. Lancet.

[4] Rittmeyer A, Barlesi F, Waterkamp D, Park K, Ciardiello F, von Pawel J, Gadgeel SM, Hida T, Kowalski DM, Dols MC (2017). Atezolizumab versus docetaxel in patients with previously treated non-small-cell lung cancer (OAK): a phase 3, open-label, multicentre randomised controlled trial. Lancet.

[5] Reck M, Rodriguez-Abreu D, Robinson AG, Hui R, Csoszi T, Fulop A, Gottfried M, Peled N, Tafreshi A, Cuffe S (2016). Pembrolizumab versus chemotherapy for PD-L1-positive non-small-cell lung Cancer. N Engl J Med.

[6] Robert C, Schadendorf D, Messina M, Hodi FS, O'Day S, investigators MDX (2013). Efficacy and safety of retreatment with ipilimumab in patients with pretreated advanced melanoma who progressed after initially achieving disease control. Clin Cancer Res.

[7] Pollack MH, Betof A, Dearden H, Rapazzo K, Valentine I, Brohl AS, Ancell KK, Long GV, Menzies AM, Eroglu Z, et al. Safety of resuming anti-PD-1 in patients with immune-related adverse events (irAEs) during combined anti-CTLA-4 and anti-PD1 in metastatic melanoma. Ann Oncol. 2018;29(1):250–55. 10.1093/annonc/mdx642 PMC5834131 29045547

[8] Spain L, Walls G, Messiou C, Turajlic S, Gore M, Larkin J (2017). Efficacy and toxicity of rechallenge with combination immune checkpoint blockade in metastatic melanoma: a case series. Cancer Immunol Immunother.

[9] Lebbe C, Weber JS, Maio M, Neyns B, Harmankaya K, Hamid O, O'Day SJ, Konto C, Cykowski L, McHenry MB (2014). Survival follow-up and ipilimumab retreatment of patients with advanced melanoma who received ipilimumab in prior phase II studies. Ann Oncol.

[10] Chiarion-Sileni V, Pigozzo J, Ascierto PA, Simeone E, Maio M, Calabro L, Marchetti P, De Galitiis F, Testori A, Ferrucci PF (2014). Ipilimumab retreatment in patients with pretreated advanced melanoma: the expanded access programme in Italy. Br J Cancer.

[11] Riaz N, Havel JJ, Makarov V, Desrichard A, Urba WJ, Sims JS, Hodi FS, Martin-Algarra S, Mandal R, Sharfman WH (2017). Tumor and microenvironment evolution during immunotherapy with Nivolumab. Cell.

[12] Naidoo J, Wang X, Woo KM, Iyriboz T, Halpenny D, Cunningham J, Chaft JE, Segal NH, Callahan MK, Lesokhin AM (2017). Pneumonitis in patients treated with anti-programmed death-1/programmed death ligand 1 therapy. J Clin Oncol.

[13] Haratani K, Hayashi H, Chiba Y, Kudo K, Yonesaka K, Kato R, Kaneda H, Hasegawa Y, Tanaka K, Takeda M, et al. Association of Immune-Related Adverse Events with Nivolumab Efficacy in non-small-cell lung Cancer. JAMA Oncol. 2017; 10.1001/jamaoncol.2017.2925 PMC6583041 28975219

[14] Postow MA, Chesney J, Pavlick AC, Robert C, Grossmann K, McDermott D, Linette GP, Meyer N, Giguere JK, Agarwala SS (2015). Nivolumab and ipilimumab versus ipilimumab in untreated melanoma. N Engl J Med.

[15] Sankarapandian V, Rehman SM, David KV, Christopher P, Ganesh A, Pricilla RA (2014). Sensitizing undergraduate medical students to consultation skills: a pilot study. Natl Med J India.

[16] Spigel DR, McLeod M, Hussein MA, Waterhouse DM, AU-Einhorn L, Horn L, Creelan B, Babu S, Leighl NB, Couture F, et al. Randomized results of fixed-duration (1-yr) vs continuous nivolumab in patients (pts) with advanced non-small cell lung cancer (NSCLC). Ann Oncol. 2017;28(suppl_5). 10.1093/annonc/mdx380.002

